# Skin microbiome and dermatologic disorders

**DOI:** 10.1172/JCI184315

**Published:** 2025-02-03

**Authors:** Tiffany C. Scharschmidt, Julia A. Segre

**Affiliations:** 1 Department of Dermatology, University of California, San Francisco, San Francisco, California, USA.; 2 Microbial Genomics Section, National Human Genome Research Institute, National Institutes of Health, Bethesda, Maryland, USA.

## Abstract

Human skin acts as a physical barrier to prevent the entry of pathogenic microbes while simultaneously providing a home for commensal bacteria and fungi. Microbiome sequencing studies have demonstrated the unappreciated diversity and selectivity of these microbes. Functional studies have demonstrated the impact of specific strains to tune the immune system, sculpt the microbial community, provide colonization resistance, and promote epidermal barrier integrity. Recent studies have integrated the microbiome, immunity, and tissue integrity to understand their interplay in common disorders such as atopic dermatitis. In this Review, we explore microbiome shifts associated with cutaneous disorders with an eye toward how the microbiome can be mined to identify new therapeutic opportunities.

## Introduction

Given the cost of maintaining sterility across the vast surface area of the skin (1), it’s no wonder that humans instead coopted beneficial microbes to colonize this exterior surface. In exchange for host-derived nutrients, such as proteins and lipids, resident skin-associated microbes prevent transient pathogenic microbes from colonizing, tune the immune system, and promote skin barrier integrity and wound repair (2, 3). Given the scarcity of resources and harsh conditions encountered, the microbial community is rather sparse on the skin surface, with greater representation in more protected niches, such as hair follicles and sebaceous glands. Dermatologic disorders managed with antimicrobial agents hint at microbial dysbiosis’ contribution to disease initiation and persistence. Here, we consider the skin-associated microbial composition and its interactions with the host immune system in the context of human disease with an eye toward future interventions.

## Uncovering the compositional microbiome of healthy skin

### Introducing the major microbial players.

**Figure 1:**
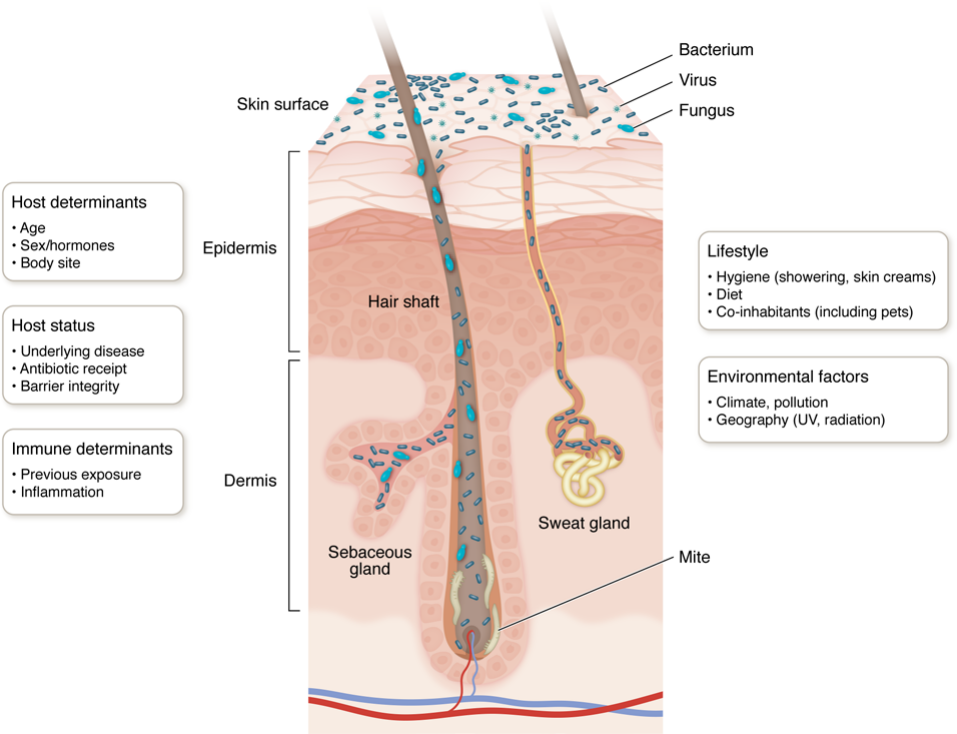
Schematic of skin histology including appendages and microorganisms. Endogenous and exogenous factors contributing to variation in the human skin microbiome.

Attention has shifted in recent decades to commensal microbes as a source of protection from transiently encountered pathogens. A limited repertoire of bacteria, fungi, viruses, and microscopic organisms colonize human skin, as most microbes are inhibited from attaching to or prospering on this external surface. Three bacterial phyla, Actinobacteria, Firmicutes, and Proteobacteria, predominate on skin, particularly commensal species *Corynebacterium tuberculostericum, Cutibacterium acnes,* and *Staphylococcus epidermidis* (4) (Figure 1). *C*. *acnes* is found at higher levels in highly sebaceous, “oily” body sites such as the forehead, chest, and back, where it resides deep within pilosebaceous units and closer to its nutrient source of sebum, a waxy oily substance that moisturizes the skin. As a facultative anaerobe, *C*. *acnes* prospers in this hypoxic environment, where it benefits the host by expressing lipases to further break down the lipid-rich sebum and release fatty acids. By contrast, *C*. *tuberculostericum* and *S*. *epidermidis* are both able to tolerate the acidic, high-salt environment produced by sweat gland secretions as well as tune their metabolism to utilize the limited nutrients available when the stratum corneum undergoes desquamation (3). Consequently, *C*. *tuberculostericum* and *S*. *epidermidis* are more prominent on the surface of moist creases and dry flexural skin. Malassezia species are the most abundant fungi on human skin, with the greatest relative abundance in sebaceous sites (5, 6). Greater fungal diversity is found on feet, consistent with the high prevalence of fungal-associated disorders such as athlete’s foot and toenail infections. Other eukaryotic organisms such as demodex mites reside within hair follicles and sebaceous glands. Finally, both prokaryotic and eukaryotic viruses are found across human skin, with Caudovirales phage and human papilloma viruses, respectively, as the most abundant species (6, 7).

### Technological advances bring new biological insight.

**Figure 2:**
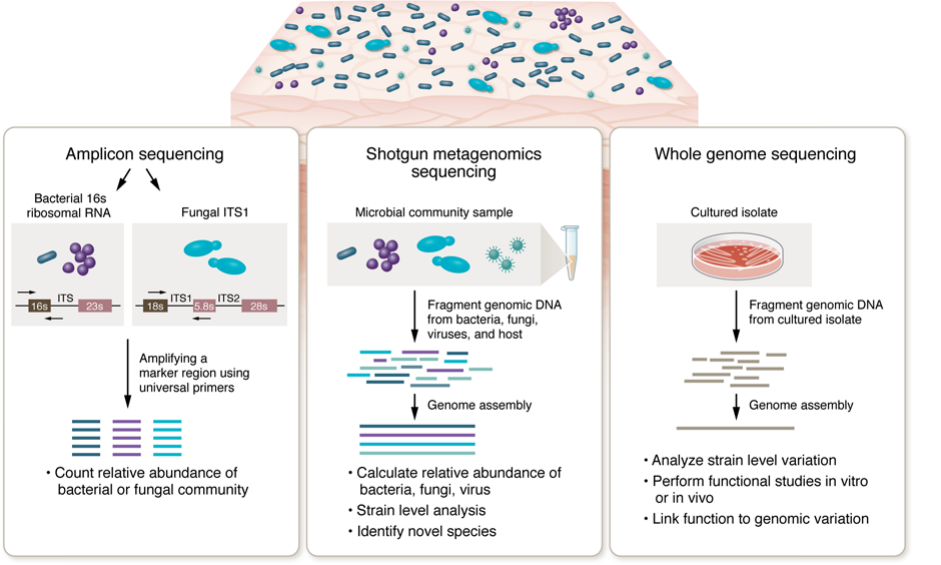
Methods to interrogate skin microbiome and avenues of downstream analysis. Swabbing the skin surface with a mild detergent to lift off the outer dead layers of skin and release the associated microbes has become the accepted best method to sample the microbiome (171). While some microbes have deeper reservoirs within the skin tissue, for example, within deeply embedded pilosebaceous units, swabs are much more tolerable to research subjects than biopsies for repeatedly sampling of the skin microbiota. After extracting DNA directly from the clinical sample, bacterial and fungal members of a skin community are identified by amplifying and sequencing 16S rRNA and ITS1 marker genes, respectively. 16S rRNA primers used to characterize the gut bacteria community failed to amplify Cutibacterium, a stumbling block rectifiable by amplifying the more 5′ region of the 16S rRNA (171, 172). Methods to make a sequencing library from the low biomass of a skin swab DNA opened up the possibility to perform skin shotgun metagenomic sequencing. After subtracting the human reads, shotgun metagenomic sequencing provides multikingdom (bacteria, fungi, virus) as well as strain-level analyses (6). The skin’s low microbial biomass created some challenges associated with air and reagent contaminants that could be monitored and controlled for with appropriate negative controls. Culturing isolates and performing whole-genome sequencing provides validation for strain level predictions and importantly provides resources for performing functional studies. Skin surface-associated bacteria persist by lowering their replication rate and perhaps even appearing as “quiescent” (173). Finding *C*. *acnes* in both cultures and shotgun metagenomic samples obtained by swabbing oily skin surfaces suggests that these microbes transit to the skin surface with terminally differentiating keratinocytes.

Advances in genomic sequencing and bioinformatic analyses have greatly enhanced our understanding of the entirety of skin microbial populations (Figure 2). Initial studies were performed by amplifying and sequencing 16S rRNA and ITS1 marker genes of bacteria and fungi, respectively. Skin shotgun metagenomic sequencing, by which all genomic material obtained in a clinical sample is directly sequenced, provided interkingdom and strain-level analyses as well as the first look at skin DNA viral populations, which lack a common marker gene and thus were not captured with amplicon sequencing (6). Moreover, combining culturing and shotgun metagenomic sequencing revealed that multiple strains of *S*. *epidermidis* and *C*. *acnes* were simultaneously present and retained over a period of years (8). Taking advantage of the topographic distribution of facial skin, Conwill and Lieberman transferred comedome contents onto agar plates to interrogate the bacterial residents of discrete pores (9). While multiple *C*. *acnes* strains were present on an individual, each pore contained a single strain, demonstrating how anatomy can constrain the mixing and coexistence of multiple subtypes. Zhou and Oh explored *S*. *epidermidis* strains and found similar strain specificity at an individual scale (10). Further investigations into metabolic diversity at the strain level will be essential to understanding host-microbe interactions and designing microbial therapeutics targeted to the skin (11, 12).

A rarely mentioned limitation of early shotgun metagenomic analyses was that the genome database used to assign reads to a microbial species was skewed towards pathogens versus commensals and, by definition, devoid of uncultivable microbes. A breakthrough came when computational biologists developed methods to de novo assemble metagenomic assembled genomes (MAGs) directly from shotgun metagenomic data and thus characterize previously uncultured species. The MAG pipeline assembles reads into contiguous (contigs) fragments based on overlapping sequences and then bins contigs into genomes based on features such as guanine/cytosine (GC) content, tetranucleotide frequency, and relative abundance in the original sample (13). By analogy, the MAG method is like being handed a box containing the intermingled pieces of a hundred different puzzles and successfully assembling each puzzle from its pieces after realizing you can sort them by color, piece size, and hinge type. Skin MAG assembly revealed novel bacterial and fungal species present in many individuals at relatively high levels that had not been cultivated, as well as novel jumbo phage. Beyond providing a new landscape of the human skin microbiome, MAGs offer a way to identify novel microbes colonizing diverse human populations over a lifespan, including patients with skin disease.

Beyond sequencing, other nascent methods have the potential to more directly ascertain the functional interactions of skin microbes with their hosts (14). Metabolomics and metaproteomics would interrogate the small molecules and proteins produced and modified by both host and microbial cells that may modulate niche colonization, immune responses, and tissue remodeling (15, 16).

### Skin microbiome across the lifespan and global diversity.

Skin microbiome evolution over the course of a human lifetime has been explored in the context of healthy, developing, and aging skin, as well as in disorders that manifest at specific stages, ranging from cradle cap of infants to acne of teenagers to xerosis of the elderly. The first sampling of vaginally born newborns revealed colonization with Lactobacillus, Prevotella, or Sneathia species transferred during passage. In infants born by cesarian section, skin commensals such as Staphylococcus, Streptococcus, Corynebacterium, and Cutibacterium species predominate (17). While both modes of delivery appear to share similar microbial profiles within a few months, there has been much consideration as to whether and how to purposefully colonize infants to maximize the health benefits provided by early colonizers or to restore the burgeoning population after antibiotic receipt (18, 19). The first year of life is full of microbial exposures as the baby transitions from being held to crawling to walking, and dramatic microbial shifts occur in site-specific ways (20). During puberty, sex hormones drive secondary maturation of sebaceous glands, which promotes sebum production and the expansion of lipophilic *C*. *acnes* and Malassezia species (21). Skin microbiome sampling of older individuals, both community-dwelling and nursing home residents, have found that functional and taxonomic microbial features more strongly associate with frailty than chronological age (22).

Examination of patients with congenital disorders has started to reveal some principles regarding the effects of host genetics. For example, studying individuals with primary immune deficiencies who have associated skin disorders has hinted at the selective shield immune surveillance provides to exogenous microbial colonization. Specifically, patients with dedicator of cytokinesis 8 (DOCK8) deficiency have increased cutaneous warts and are extensively colonized by human papillomaviruses (23), whereas those with recombination-activating gene (RAG) deficiency displayed increased nasal colonization with RNA viruses and ecological permissiveness. Similarly, Netherton patients with mutations affecting skin barrier integrity exhibit perturbed skin microbiome communities, colonized by strains of *Staphylococcus aureus* and *Staphylococcus epidermidis* that promote skin inflammation (24).

A year-long study of healthy adults not exposed to antibiotic treatments illustrated that composition of an individual’s skin microbiome is relatively stable over time (8). While additional work is clearly needed to rigorously study a fuller range of exposures that might perturb the skin microbiomes, early studies do indicate that commonly prescribed oral antibiotics can have persistent effects on skin microbiome composition and antimicrobial resistance genes (25, 26).

Another important consideration of skin microbiome studies is geographic diversity, a key limitation of the field with the majority of studies focused on industrialized populations (27). One amplicon study of indigenous Amerindians demonstrated a skin microbiome with greater bacterial and genetic diversity (28), stressing the importance of additional studies to understand how genetic diversity as well as social and cultural norms influence the composition of skin microbiota populations.

## Physiologic functions of the skin microbiome

### The skin microbiome in barrier defense.

As a first line of defense in colonization resistance, commensal microbes occupy space and resources at the skin surface. Decades of research have also focused on the small commensal-derived molecules and proteins that directly antagonize invading pathogens, as best studied for *S*. *aureus* (29). Early studies discovered that a subset of commensal *S*. *epidermidis* strains secrete the serine protease Esp to hinder *S*. *aureus* nasal colonization (30). Several additional antimicrobial peptides produced by coagulase negative *Staphylococcal* species have been identified, including epidermin (31), lugdunin (32), and shA9, which is currently undergoing trials for clinical efficacy (33). The thiopeptide cutimycin was bioinformatically mined from *C*. *acnes* genomes and shown to limit *S*. *aureus* colonization (34). Beyond prevalent skin microbial species, additional microbes such as *Bacillus subtilis* and *Roseomonas mucosa* have also been tested to assess whether they diminish *S*. *aureus* colonization (35, 36). In addition to killing or reducing growth of their neighbors, skin bacteria also secrete molecules that can modify behavior of surrounding species via quorum-sensing systems. For example, coagulase-negative *Staphylococci* (CoNS) produce autoinducing peptides which can inhibit activity of the accessory gene regulator (agr) quorum-sensing system of *S*. *aureus*. This *agr* regulation in turn reduces *S*. *aureus* production of proinflammatory toxins and virulence factors detrimental to the skin barrier (37, 38).

Murine models have also demonstrated how skin microbes can enhance skin-barrier function. For example, a sphingomyelinase produced by *S*. *epidermidis* helps bacteria to acquire essential nutrients while also promoting keratinocyte production of ceramides, a major component of lamellar structures (39). Transcriptional profiling of the skin of germ-free mice before and after microbial colonization demonstrated changes in biological functions associated with skin development and differentiation. While many epidermal structures are intact in the skin of germ-free mice, close examination showed the microbiota promotes epidermal differentiation and barrier integrity through keratinocyte signaling of the aryl hydrocarbon receptor (40). New in vitro models to assess host-microbial interactions include growing microbes in defined human sweat media (41) at the air-liquid interface (42). Clinically relevant models to assess both individual strains and consortium of microbiota will greatly benefit the field as it moves to more mechanistic studies of host-microbe interactions.

### Innate immunes responses to the skin microbiome.

**Figure 3:**
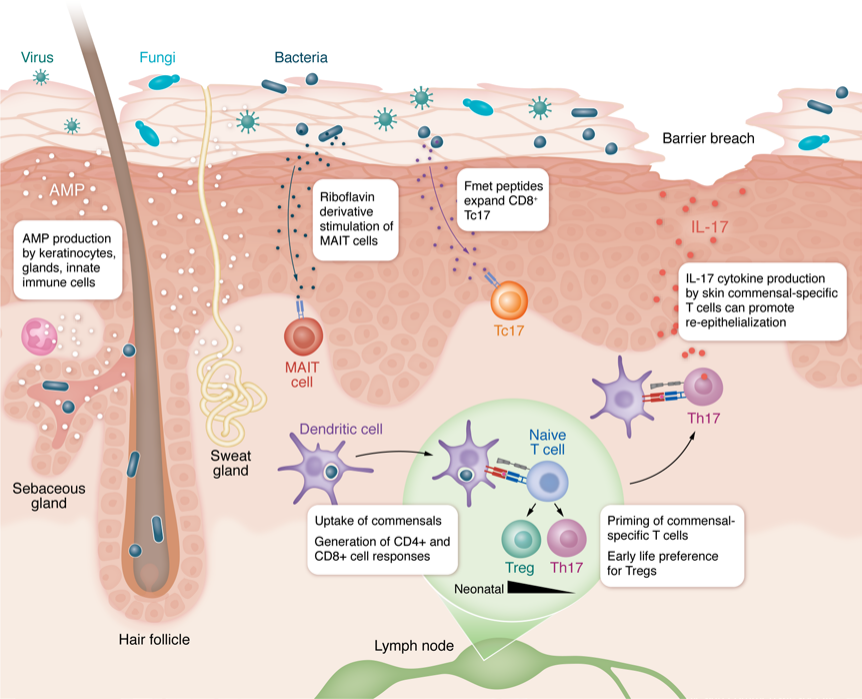
Commensal tuning of cutaneous immune function. Commensal microbes stimulate production of antimicrobial peptides (AMPs) by keratinocytes, sebocytes, sweat glands, and granulocytes, amplifying the innate immune defense of skin against potential pathogens. Specific microbial products also have the ability to expand certain skin lymphocyte populations. For example, riboflavin derivatives made by various bacteria are recognized by skin MAIT cells leading to their expansion and activation in the tissue. Similarly, fmet peptide produced by a particular clade of *S*. *epidermidis* is presented by nonclassical MHC-I molecules and leads to expansion of IL-17–producing CD8^+^ T cells (Tc17). DCs play a role both in initial uptake of skin bacteria and presentation of commensal antigens to naive T cells in the skin-draining lymph node as well as restimulation and cytokine production by commensal-specific skin-resident T cells. Type 2 DCs are especially capable of commensal uptake, at least for *S*. *epidermidis*, as shown in human and murine skin. In the early life window, these DC2 also play a key role in preferentially generating commensal-specific Tregs versus conventional T cells, usually Th17 cells. Commensal-specific Th17, however, also plays a key role in immune defense as well as homeostatic functions such as reepithelialization after barrier breach.

Innate immunity provides an additional layer of “molecular and cellular” protection to our skin’s physical barrier (Figure 3). Studies in mice have illustrated that skin microbes stimulate antimicrobial peptides production by keratinocytes and sebocytes (43–45). Keratinocytes respond to TLR stimulation, or sensing of cell injury due to bacterial proteases and toxins, by secreting cytokines that can recruit and activate immune cells (46–48). TLR sensing of *Staphylococcus spp*. has also been shown in mice to reactivate specific endogenous retroviruses (ERV) in keratinocytes, thereby augmenting cutaneous cGas/STING signaling and type 1 interferon production (49). Despite conserved epitopes between commensal and more prototypically pathogenic species, the amount and sometimes quality of cutaneous cytokine production elicited by commensals can be quite distinct, for example, with less IL-1 family cytokines produced in response to *S*. *epidermidis* than *S*. *aureus* colonization (50, 51). Although commensal microbes usually succeed in promoting homeostatic immunity without eliciting overt skin inflammation, this balance can be tipped towards pathology in certain contexts, for example, deficient epidermal barrier function or high bacterial loads (24, 52–54).

Capture of microbial antigens by antigen-presenting cells (APCs) is a critical initial step toward generating bacteria-specific immune memory. Skin is replete with epidermal macrophages, i.e., Langerhans cells, as well as dermal macrophages and conventional DCs (cDCs). While all of these APCs are capable of phagocytosing skin commensal bacteria, type 2 cDCs (cDC2s) and especially those that express the C-type lectin CD301b are highly efficient at capturing bacteria in murine and human skin as well as trafficking bacterial antigens to the skin-draining lymph nodes in mice (55). In healthy mouse skin, these CD301b cDC2s preferentially support generation of commensal-specific Tregs (55). By contrast, type 1 cDCs facilitate antigen-specific CD8^+^ T cell responses to *S*. *epidermidis* (56). While most microbial antigens are presumably sensed by classical MHC molecules on APCs, some bearing N-formylated methionine are recognized by nonclassical MHC-I molecules. These include fMet peptides produced by a small clade of *S*. *epidermidis* that elicit skin CD8^+^ T cell responses in mice and nonhuman primates (57).

### Microbial tuning of adaptive immunity.

Over a decade ago, a pivotal study comparing germ-free versus *S*. *epidermidis* mono-colonized mice, illustrated the key adjuvant role that commensal bacteria play in cutaneous adaptive immunity, increasing the number of skin T cells and their cytokine production, thereby bolstering defense against infectious threats (46, 58). Since then, a body of work largely in mouse models has started to uncover the molecular basis of distinct adaptive immune responses elicited by different microbes, e.g., *S*. *epidermidis* and other CoNS (56, 57), *Corynebacterium spp* (59), and fungal symbionts (60, 61). One shared feature of these interactions is that superficial colonization of skin tends to elicit a type 17 immune response, as compared to the more type 1– or Th1-dominant response seen after intradermal skin injection (56, 60). These IL-17–producing, commensal-specific CD4^+^ and CD8^+^ T cells augment cutaneous defense against pathogens, while simultaneously they are capable of producing type 2 cytokines and contributing to reepithelialization following skin injury (57, 62).

Healthy skin contains many T cells but fewer B cells. Thus, most studies have focused on commensal tuning of T cell responses. However, recent work has illuminated that the skin, like the gut, is a site for initiation of humoral antibody-mediated immunity to commensal microbes. Topical association of mice with *S*. *epidermidis* was sufficient to expand antigen-specific B cells in both the skin-draining lymph nodes and newly formed cutaneous tertiary lymphoid organs (TLOs), a process critically supported by Langerhans cells. In turn, B cells in the cutaneous TLOs preferentially secreted an *S*. *epidermis*–specific antibody of the IgG2b isoform, which helped limit the total *S*. *epi* load on the skin surface (63). Whether this mechanism contributes to microbe-immune homeostasis in healthy human skin remains to be seen, but it is interesting to consider in the context of skin diseases where significant B cell infiltrates can be seen, i.e., cutaneous lupus and hidradenitis suppurativa (HS) (64, 65).

### Neuronal, adnexal, and stromal interplay.

Neurons, fibroblasts, and adnexal structures are also instrumental players in tissue immunity, and we are starting to understand their roles in modulating immune-microbe crosstalk in skin. For example, *S*. *aureus* can directly elicit neuronal pain and itch in murine models through the respective effects of its pore-forming toxins and proteases on peripheral neurons (66–68). *S*. *epidermidis*–specific IL-17^+^CD8^+^ T cells in murine skin have been observed to localize near nerve endings and to preferentially express *Ramp1*, a receptor for the neuropeptide CGRP. Indeed, T cell–intrinsic sensing of neuron-derived CGRP in mouse skin was sufficient to constrain the total numbers of commensal-specific T cells as well as their degree of activation (69). Our knowledge of how stromal cell populations contribute to microbiota-directed immune responses is likewise growing, especially in regard to microbial infiltration of the dermis. Dermal adipocytes and preadipocytes can be important sources of antimicrobial peptides (15, 70), while fibroblasts can promote neutrophil recruitment via release of CXCL12 and other ligands following IL-17 sensing (71).

Hair follicles constitute a major immune niche in the skin as well as a reservoir of commensal microbes (72), and as such are a major area of focus for studies in mice trying to dissect cutaneous microbe-immune interactions. In healthy skin, hair follicle keratinocytes produce cytokines that promote survival and perifollicular localization of skin T cells (73). Neonatal colonization of hair follicles by commensal microbes can augment this cytokine production and by extension facilitate recruitment of Tregs into developing mouse skin (74). Activity of sebaceous glands, found most often in conjunction with hair follicles as part of the pilosebaceous unit, sustains survival of lipotrophic skin bacteria by providing essential nutrients (75). Growth of sebaceous glands is itself regulated by perifollicular skin-resident innate lymphocytes that rely on cytokine signals produced by hair follicles (76). Although germ-free mice demonstrated reduced sebaceous gland activity, microbial association was insufficient to immediately rescue this phenotype. Instead, rescue required transgenerational effects, illustrating the complexity of host-microbe crosstalk at the skin barrier (77).

### Early life interactions.

Neonatal life represents a dynamic period for both host immune development and assembly of commensal communities (20, 78). It is also a critical window for establishment of adaptive immune tolerance (79). As such, early life perturbations of immune-microbe interactions can have longstanding consequences for the host (80), and it should come as little surprise that this period is denoted by some distinct immune responses to skin commensal colonization. The first of these relates to adaptive immune tolerance and the generation of commensal-specific Tregs. Using a model antigen to track generation of *S*. *epidermidis–*specific CD4^+^ T cells responses in mice, colonization of neonatal versus adult skin has been shown to generate a much higher proportion of Foxp3^+^ *S*. *epidermidis*–specific Tregs (81, 82). This enriched population of commensal-specific Tregs can then protect the skin against inflammation upon reexposure to the same commensal during subsequent skin injury in adulthood (82). High proportions of polyclonal Tregs in developing skin (82) as well as enhanced retinoic acid production by neonatal versus adult skin CD301b^+^ cDC2 (55) are both mechanisms identified thus far that contribute to the preferential early window for tolerance.

Generation of mucosal-associate invariant T (MAIT) cells, which constitute a substantial portion of T cells in both human and murine skin, represents another age-restricted interaction between the skin microbiome and cutaneous immune system (83). MAIT cells are denoted by expression of an oligoclonal T cell receptor (TCR) repertoire and can be stimulated either through antigen recognition or direct cytokine sensing, thus spanning aspects of innate and adaptive immune function (84). Riboflavin derivatives produced by intestinal and skin bacteria are a known ligand for MAIT TCRs (85, 86). Notably, thymic generation of MAIT cells in response to riboflavin-producing bacteria has been shown to require the presence of these microbes specifically during the neonatal window (83). Subsequently, the presence of riboflavin-producing bacteria at the skin barrier can promote local expansion of MAIT cells in the tissue as well as their production of IL-17. These cells can then contribute to skin homeostasis through various functions shared with other IL-17–producing skin T cells, such as host defense and epithelial repair (83).

## The skin microbiome in disease

In parallel with efforts to uncover the composition and physiologic function of the skin microbiome in health, there has been robust investigation into how microbial shifts contribute to human disease. As noted above, the topographical diversity of the skin microbiome across body sites and its sensitivity to perturbation by topical or systemic antimicrobial agents (25) add complexity to study design along with inherent challenges in considering “chicken or the egg” questions. Even so, our understanding of how the microbiome might contribute to pathophysiology of various skin diseases has advanced in recent years.

### Atopic termatitis.

Atopic dermatitis (AD) is a chronic inflammatory disorder characterized by episodic flares of itchy eczematous lesions, in which epidermal barrier impairment and immune dysregulation play important roles. However, the consistent bacterial alterations accompanying disease flares and the clinical response to antimicrobials make AD a quintessential skin disorder to dissect the role of the skin microbiome (87). AD prevalence is approximately 20% of children, but also affects adults. Early studies implicated the “hygiene hypothesis” linking decreased AD risk with lifestyle factors associated with increased microbial exposure such as dog ownership, farm residence, daycare attendance, or older siblings (88). Over fifty years ago, culture-based studies identified increased *S*. *aureus* skin abundance on AD lesional and nonlesional sites (89). More recent sequencing-based studies have consistently confirmed increased *Staphylococcal* prevalence during AD flares as well as a relative reduction following effective treatment with topical steroids or dupilumab (90–92). Specifically, *S*. *epidermidis* increases during flares in young moderately affected children, with *S*. *aureus* seen in older children and more severe disease (90, 93). As AD resolves in 70% of children by the age of seven, it would be fascinating to follow a birth cohort of AD infants to understand how their flare-associated microbiome changes with age and if it serves as a marker for a subtype of AD treatment response or long-term outcome. Given shifts in the microbiome observed between baseline and flare, it would also be interesting if we could use fluctuation dynamics of the skin microbiome to predict when a child was about to flare, allowing earlier treatment and reduction of skin inflammation. Given slow turnaround for sequencing analysis, metabolic signatures of specific flare-associated microbes are an intriguing alternative target. Addressing the frequency and severity of flares would have immediate benefit to affected children and with potential long-term prevention of other atopic conditions, including asthma and allergic rhinitis.

Two critical questions are at the forefront of the AD skin microbiome field. First, does the skin microbiome, and specifically *Staphylococcal* species, drive disease? Distinct *S*. *aureus* strains have been associated with AD and disease severity with some geographic specificity, perhaps due to differential prescribing practices of antibiotics (87). Skin colonization of mice with AD-associated *S*. *aureus* strains has been shown to mimic features of AD, including epidermal thickening and immune cell infiltration (51, 90), mast cell degranulation (94), and eosinophilia (95). AD-associated *S*. *aureus* strains can also produce proteases that impair skin barrier integrity, while concomitantly activating neurons to induce itch (68). The second question is whether the skin microbiome of healthy children provides some protection either at the individual strain or community level. Starting at age two to three months, higher levels of commensal Staphylococcus was associated with lower likelihood of developing AD at age two (96, 97). This fits into a model where early development of immune tolerance to skin commensals is important for protection against later inflammation. However, animal studies have suggested that barrier dysfunction may undermine early life establishment of commensal-specific Tregs (54). As discussed above, several commensal bacterial strains shown to have antimicrobial effects against *S*. *aureus* have been identified as deficient in AD skin (98), raising the possibility of mining and harnessing endogenous microbiota for therapeutic molecules and behaviors. As further discussed below, AD is the disease with the most clinical trials approved to test safety and efficacy of such live microbial therapeutics.

### Acne vulgaris.

Acne vulgaris has long been linked to *Cutibacterium acnes*, a prevalent member of the healthy, postadolescent skin microbiome, especially at sebaceous skin sites commonly affected by acne, such as the face, chest, and back (21, 99). Sequencing of the acne-associated microbiome has shown largely equivalent relative abundance of *C*. *acnes* as compared with age-matched healthy volunteers (100–102). However, sampling of individual lesions within affected subjects has revealed increased *C*. *acnes* prevalence in inflammatory versus comedonal (whitehead) lesions (103). Culture-based studies indicate that *C*. *acnes* levels in a single acne comedone approach those found over an entire square centimeter of healthy facial skin (104). Metagenomic sequencing has revealed particular *C*. *acnes* strains (100, 101, 105, 106) and certain metagenomic features (107) to correlate with disease. These features include enrichment in genes encoding particular metabolic pathways (100) as well as putative virulence elements, such as antimicrobial peptides, cytotoxins, and proteases (107). Ex vivo proteomic and transcriptomic analysis of *C*. *acnes* strains isolated from acne skin also support functional metabolic differences (106). This suggests that certain *C*. *acnes* isolates may be adapted to survival in the inflammatory acne environment and likewise contribute to its pathogenesis. There are two limitations of these natural history acne studies: first, they rarely follow subjects through the onset of acne, and second, recruited cohorts are typically years or decades past the initial age of disease onset, making it hard to untangle whether observed strain selectivity is strictly correlated with disease or results from prior antibiotic treatment. Ex vivo functional assays have shown that acne-associated isolates are capable of eliciting heightened responses from human immune cells (108, 109), keratinocytes (110), and sebocytes (105). Thus, recent work in the area of the acne microbiome has added both clarity and complexity to our understanding of how bacterial species and strains are correlated with disease pathogenesis.

### Alopecia.

Alopecia encompasses a set of hair loss disorders, segregated into scarring and nonscarring entities based on the presence or absence of inflammatory hair follicle destruction. Certain scarring alopecias, denoted by neutrophilic infiltrates, have long been associated with microbial infiltration of the hair follicle, for example, by *S*. *aureus* (111). Sequencing-based approaches have added some additional nuance to our understanding of disease-associated shifts in the microbiome in these (112) and other lymphocyte-rich scarring alopecias (113). However, most recent research has focused on microbiome contributions to the autoimmune nonscarring condition, alopecia areata (AA). This has included examination of intestinal microbiome composition as a potential source of altered immune function, which has yet to reveal consistent disease-associated changes (114–116). Other 16s ribosomal DNA (rDNA) sequencing studies of the scalp microbiome suggest that AA is accompanied by a relative increase in Proteobacteria, such as *Corynebacterium* and *Cutibaterium spp*, with a concurrent decrease in CoNS (117–119). Whether these shifts are instrumental to disease onset and/or severity or a consequence of disease- or therapy-associated changes to the skin environment remains undetermined.

### HS.

HS is characterized clinically by inflammatory nodules and sinus tracks that predominantly affect flexural skin sites. Early stages of disease respond, at least partially, to microbe-directed therapies such as topical and oral antibiotics. Research on HS has encompassed both the skin and gut microbiome (120–123). There is rationale for examining the intestinal microbiome in HS given its coassociation with inflammatory bowel disease (124), but here we will focus on key features of the skin microbiota. HS presents a particular challenge for skin microbiome sampling, as the stage and morphology of lesions can vary significantly within a given individual, and many lesions are characterized by deep, subcutaneous inflammation. Skin microbiome studies of HS have, thus, employed not only standard skin swab sampling (125, 126), but also skin biopsies (127, 128). Results from 16S rDNA sequencing have shown with relative consistency that affected HS skin, and particularly deeper lesions, have increased relative abundance of anaerobic bacteria, such as *Prevotella, Porphymonas, Fusobacteria,* and *Bacteroides,* and reduced actinobacteria, such as *Corynebacterium* and *Cutibacterium spp* (126–128). Clinically unaffected areas of skin from HS subjects also demonstrate similar but less pronounced changes (125, 126). It will be exciting to see results from ongoing work in this area, which will hopefully include metagenomic sequencing approaches, functional assessment of disease-associated strains, and diverse research subjects reflective of the demographics most often affected by HS (129).

### Psoriasis.

Unlike AD, HS, or acne vulgaris, psoriasis is not typically treated with antimicrobial agents. However, the guttate form of psoriasis can be triggered by preceding *Streptococcal* infection. This observation along with the high prevalence and chronicity of plaque-type psoriasis have inspired a search for microbiome-based contributions to its pathogenesis. While there have been few reports of increased Firmicutes, studies have not identified a consistent psoriasis skin microbiome signature (130–132). 18s rDNA sequencing and qPCR of psoriatic skin lesions have shown a relative and absolute increase in *Malassezia spp*. (133, 134). Notably, in a small study performed several decades ago, topical application of heat-killed *Malassezia ovalis* or *S*. *epidermidis* elicited psoriatic lesions in patients with preexisting psoriasis (135). This has been corroborated by murine studies looking at the effects of commensal fungi and bacteria in models of psoriasiform skin inflammation (59, 60). Work examining the gut microbiome of psoriasis patients has unveiled some consistent disease-associated shifts, such as a relative increase in *Firmicutes* and *Actinobacteria* and a decrease in *Bacteroides* (136–140). Considering that psoriasis encompasses a heterogeneous clinical disease spectrum, it is perhaps not surprising that some studies have identified distinct gut “enterotype” profiles among psoriasis subjects, which in certain cases also correlated with clinical subtypes or disease severity (136, 141, 142). A team employing a Mendelian randomization approach to explore a causal link for the gut microbiome in over 18,000 psoriasis patients identified a handful of gut microbiome features with a small but statistically significant change in psoriasis risk (143). Moving forward, larger studies and those focusing on specific forms of psoriasis (e.g., guttate psoriasis) may be needed to define if the skin and/or gut microbiome contribute to psoriasis pathogenesis in at least some individuals.

### Seborrheic dermatitis.

Seborrheic dermatitis is another chronic inflammatory condition, primarily affecting the scalp, face, and chest, in which shifting skin microbiome composition, specifically of *Malassezia spp*. and the mycobiome, is thought to play a pathogenic role. Indeed, antifungal shampoos and creams are a treatment mainstay. 18s rDNA sequencing has confirmed the findings of culture-based studies that indicated a role for *Malassezia* in seborrheic dermatitis (47). However, analogous to acne vulgaris and *C*. *acnes*, further work is needed to disentangle how this ubiquitous commensal yeast contributes to seborrheic dermatitis. In parallel, 16S rDNA sequencing has revealed increased prevalence of coagulase-negative *Staphylococcus spp*. (47, 144, 145). Considering that antibacterial treatments are not readily used or effective in seborrheic dermatitis, it is quite possible that this shift in skin bacteria is a secondary change following disease onset.

### Skin ulcers and wounds.

The skin microbiome’s influence on wound healing and clinical outcomes of skin wounds and ulcers was recently reviewed in depth (146). Upon injury, the skin microbiota composition can shift (147), which has the potential to facilitate or impede the healing process (148). Commensal microbes, including certain *S*. *epidermidis* isolates, have been shown to promote wound healing through mechanisms including immune modulation, barrier function enhancement, and modulation of matrix metalloproteinase 10 (40, 57, 83, 149, 150). Conversely, *S*. *aureus* and *Pseudomonas aeruginosa* can lead to chronic inflammation, delayed healing, and persistent ulcers (151, 152). Understanding the balance between beneficial and harmful microorganisms is essential for developing microbiome-targeted therapies. Emerging approaches to optimize the wound microbiome and promote repair, including but not limited to live microbial transplant (153), will hopefully offer new options.

### Posttransplant immune responses.

Solid organ and stem cell transplantation represent major advances of modern medicine, but hurdles remain. For example, graft-versus-host disease (GVHD) manifesting as inflammation at barrier sites such as the skin or intestine complicates many stem cell transplants. Separately, while autologous skin grafting is a commonly used surgical practice, successful transplantation of nonautologous skin, e.g., facial transplant, is less common and still has a high rate of rejection (154). Whether skin microbes contribute to these ongoing challenges remains uncertain, but some evidence in animal models supports this idea. For example, transplant of a *S*. *epidermidis* monocolonized skin graft hastened rejection in mice by augmenting the immune response to allogeneic antigens (155). Commensal-specific T cells also infiltrate and damage *S*. *epidermidis*–colonized syngeneic grafts (i.e., from a genetically identical donor), especially in recipient mice with preexisting commensal-specific memory T cells (156). How much anticommensal immune responses contribute to human skin inflammation in cutaneous GVHD following allogeneic stem cell transplant is a distinct and unanswered question. Although pilot studies have explored composition of the skin microbiome in cutaneous GVHD (157), it is difficult to adequately control for the role of immunosuppressive or antimicrobial medicines. Future studies that examine composition of the pretransplant skin microbiome in both donor and recipient as well as their anticommensal T cell repertoire will hopefully further inform our understanding.

## Looking ahead: therapeutic opportunities leveraging the skin microbiome

### Bugs as drugs.

**Figure 4:**
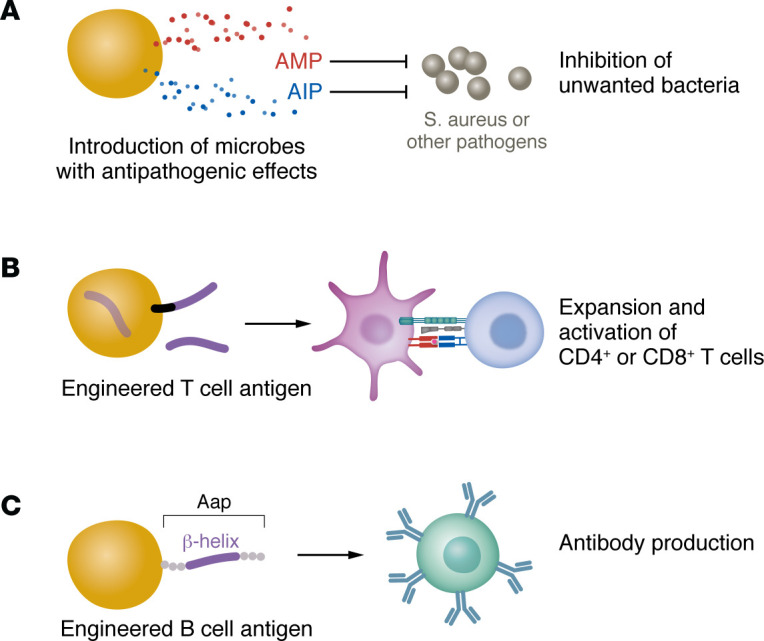
Emerging skin microbiome–based therapeutic approaches. (**A**) Current clinical trials involving live microbiome-based skin therapeutics are based on the paradigm of (re)introducing or increasing the abundance of commensal microbes that naturally produce molecules with bioactivity to combat growth or pathogenicity of unwanted bacteria such as *S*. *aureus*. These strains typically produce AMPs, which can directly kill bacteria, or autoinducing peptides (AIPs), which impede pathogenic behaviors by inhibiting quorum-sensing dependent expression of key virulence factors. (**B**) Another therapeutic approach that has recently garnered attention due to promising results in animal models, but which remains untested in humans, is using skin commensals as chassis for exogenous expression of immunogenic antigens. In mice, for example, expression of tumor antigens (purple lines) by *S*. *epidermidis* enhanced immune responses against melanoma. These could stimulate both CD4^+^ and CD8^+^ T cells through a combination of surface and secreted antigen expression. (**C**) Separately, recent work has identified the accumulation-associated protein (aap) on the surface of *S*. *epidermidis* as especially stimulatory towards B cell–mediated antibody responses. Mice colonized with a strain of *S*. *epidermidis* engineered to express a nonnative peptide in place of the central aap beta-helix (purple segment) generated antigen-specific antibodies sufficient to protect them against humoral immune response to tetanus toxin.

As detailed above, our skin microbiome encodes antibiotics, enzymes, quorum-sensing molecules, and other substances that can influence skin health directly or via effects on other microbes. Indeed, a large motivation for research in this field has been the hope that microbial products might be harnessed to prevent or treat skin disease, and in recent years, this is looking more like a tenable possibility (Figure 4). Similar to oral probiotics for gut health, growing consumer awareness of the skin microbiome has led to an increase in cosmetic products touted to “benefit the skin microbiome” through probiotic or prebiotic components. However, this is categorically different than having an FDA-approved live bacterial therapy rigorously tested for evidence of clinical benefit and safety. Since 2017, several investigational new drug applications have been approved and phase 1 and 2 trials initiated to test topical application of live bacteria to skin, the majority targeting AD. Like any area of drug development, there have been therapies that failed phase 2 (36, 158) but several live therapeutics that have shown promise (33, 159, 160) remain in active investigation (ClinicalTrials.gov NCT06504160; NCT06469385; NCT06096857). For example, TIME-2 is an ongoing randomized multicenter phase 2 trial to study topical application of *S*. *hominis* expressing the antimicrobial peptide A9 (33, 161). This builds on a previous phase 1 trial and careful studies in mice and humans. These have uncovered important insights, such as that A9 expression causes *S*. *hominis* to be more susceptible to killing by host antimicrobial peptides (162). This led to incorporation of topical steroid administration as an adjuvant treatment in TIME-2 to reduce host antimicrobial peptide production. In general, there are many things to consider in designing a live therapeutic for topical versus oral administration, the most obvious of which is formulation — will it be spray, cream, ointment? How often should it be administered for good results — daily or only during flares? And to what areas of the body — diseased skin only or more generally? The arena of bacterial therapeutics for skin disease will remain very dynamic and exciting in coming years. The approval of one or more products in this area for chronic inflammatory skin conditions, such as AD, HS, or acne, would certainly be welcomed by patients and clinicians alike.

### Harnessing skin microbes ability for immune modulation.

The potential therapeutic impact of antibacterial immune responses is not a new concept. In fact, it inspired some of the first forms of anticancer immunotherapy (163). However, growing knowledge of our commensal microbiota and the immune responses they elicit presents new opportunities to imagine how these might be leveraged for health (164). Indeed, the notion that intestinal microbial composition could predict (41) or modulate (165–167) responses to anti-PDL1 cancer immunotherapy has motivated a substantial number of clinical trials (168). However, we must also recognize where our knowledge yet falls short. We still have a limited molecular understanding of microbe-specific components that can tune cutaneous immune responses. To date, CD8-promoting fMet peptides expressed by a specific clade of *S*. *epidermidis* (57) and Th17-promoting mycolic acids produced by *Corynebacterium spp*. (59) are rare exceptions where we have gained some level of molecular understanding. Perhaps newly developed approaches to rapidly probe these interactions will accelerate knowledge in this area (16). Even so, a proximal goal might be leveraging skin commensal bacteria as chassis for antigens or other molecules with well-defined and desirable immune effects. One study elegantly demonstrated that *S*. *epidermidis* can be engineered to express melanoma antigens that augmented antimelanoma CD4^+^ and CD8^+^ T cell responses and tumor clearance in *S*. *epidermidis–*colonized mice, both within skin and at more distal sites (169). In a recent preprint (63), *S*. *epidermidis* was also shown capable of eliciting a potent host antibody response targeting its cell surface protein Aap. Leveraging click-chemistry to decorate *S*. *epidermidis* Aap with non-native antigens, the host humoral response could be redirected toward tetanus toxin with protective effects in a tetanus infection model (63). Although these animal models may not adequately reflect the complexity of preexisting human immune responses to commensal microbes, which can be distinctly shaped by early life interactions (170), using skin commensal microbes as a delivery system for immune-mediated therapies remains a very exciting possibility.

## Conclusion

The skin microbiome represents a complex and dynamic ecosystem that plays a crucial role in maintaining skin health and overall tissue homeostasis. Understanding the intricate interactions between host and microbial communities has revealed novel insights into the pathogenesis of various skin conditions and highlighted the potential for innovative therapeutic strategies. Future research must continue to explore the composition and functionality of skin-associated microbiota, particularly in diverse populations, microbiome changes accompanying skin disease onset, and their application in therapeutic contexts. By advancing our knowledge of the skin microbiome’s contributions to immune regulation, barrier function, and disease, we can pave the way for targeted interventions that promote skin health and prevent dermatological disorders. This evolving field holds great promise, offering new avenues for both basic scientific discovery and clinical application.

## Version 1 02/03/2025

Electronic publication
